# Insanity and Its Treatment

**Published:** 1872-08

**Authors:** 


*Insanity and its Treatment. Lectures on the treatment, Medical and Legal of Insane patients.* By G. Fielding Blandford, M. D., Oxon. With a summary of the laws in force in the United States, on the confinement of the insane. By Isaac Ray, M. D. Philadelphia: Henry C. Lea, 1871.

The lectures comprising this work were delivered at the school of St. George's Hospital, and hence the book is to be regarded more as a text-book for students than as a complete treatise on the subject of insanity. As such it is remarkably clear and explicit, and in the short space allotted necessarily to each lecture, treats the subjects under consideration in a very full and satisfactory manner.

The appendix will add much in value to the American edition. The author

Dr. Ray, has gained so wide and well-deserved a notoriety on the subject of insanity, that anything coming from his pen may well receive the attention of the profession. The work is one which cannot fail to impart much information to the observing and careful student, and to such we recommend it as a book highly calculated to meeet their wants in the department of diseases of the mind.

